# Association of Mindfulness-Based Interventions With Anxiety Severity in Adults With Cancer: A Systematic Review and Meta-analysis

**DOI:** 10.1001/jamanetworkopen.2020.12598

**Published:** 2020-08-07

**Authors:** Sapna Oberoi, Jiayu Yang, Roberta L. Woodgate, Saroj Niraula, Shantanu Banerji, Sara J. Israels, Gary Altman, Sara Beattie, Rasheda Rabbani, Nicole Askin, Abha Gupta, Lillian Sung, Ahmed M. Abou-Setta, Ryan Zarychanski

**Affiliations:** 1 Departments of Pediatrics and Child Health, University of Manitoba, Winnipeg, Manitoba, Canada; 2 Department of Internal Medicine, University of Manitoba, Winnipeg, Manitoba, Canada; 3 College of Nursing, University of Manitoba, Winnipeg, Manitoba, Canada; 4 Department of Medical Oncology and Hematology, CancerCare Manitoba, Winnipeg, Manitoba, Canada; 5 Department of Psychiatry, University of Manitoba, Winnipeg, Manitoba, Canada; 6 Alberta Blood and Marrow Transplant Program, Alberta Health Services, University of Calgary, Calgary, Alberta, Canada; 7 George and Fay Yee Center for Healthcare Innovation, Winnipeg Regional Health Authority, University of Manitoba, Winnipeg, Manitoba, Canada; 8 Neil John Maclean Health Sciences Library, University of Manitoba, Winnipeg, Manitoba, Canada; 9 Division of Hematology/Oncology, The Hospital for Sick Children, Toronto, Ontario, Canada

## Abstract

**Importance:**

Mindfulness-based interventions (MBIs), grounded in mindfulness, focus on purposely paying attention to experiences occurring at the present moment without judgment. MBIs are increasingly used by patients with cancer for the reduction of anxiety, but it remains unclear if MBIs reduce anxiety in patients with cancer.

**Objective:**

To evaluate the association of MBIs with reductions in the severity of anxiety in patients with cancer.

**Data Sources:**

Systematic searches of MEDLINE, Embase, Cochrane Central Register of Controlled Trials, CINAHL, PsycINFO, and SCOPUS were conducted from database inception to May 2019 to identify relevant citations.

**Study Selection:**

Randomized clinical trials (RCTs) that compared MBI with usual care, waitlist controls, or no intervention for the management of anxiety in cancer patients were included. Two reviewers conducted a blinded screening. Of 101 initially identified studies, 28 met the inclusion criteria.

**Data Extraction and Synthesis:**

Two reviewers independently extracted the data. The Cochrane Collaboration risk-of-bias tool was used to assess the quality of RCTs, and the Preferred Reporting Items for Systematic Reviews and Meta-analyses reporting guideline was followed. Summary effect measures were reported as standardized mean differences (SMDs) and calculated using a random-effects model.

**Main Outcomes and Measures:**

Our primary outcome was the measure of severity of short-term anxiety (up to 1-month postintervention); secondary outcomes were the severity of medium-term (1 to ≤6 months postintervention) and long-term (>6 to 12 months postintervention) anxiety, depression, and health-related quality of life of patients and caregivers.

**Results:**

This meta-analysis included 28 RCTs enrolling 3053 adults with cancer. None of the trials were conducted in children. Mindfulness was associated with significant reductions in the severity of short-term anxiety (23 trials; 2339 participants; SMD, −0.51; 95% CI, −0.70 to −0.33; *I*^2^ = 76%). The association of mindfulness with short-term anxiety did not vary by evaluated patient, intervention, or study characteristics. Mindfulness was also associated with the reduction of medium-term anxiety (9 trials; 965 participants; SMD, −0.43; 95% CI, −0.68 to −0.18; *I*^2^ = 66%). No reduction in long-term anxiety was observed (2 trials; 403 participants; SMD, −0.02; 95% CI, −0.38 to 0.34; *I*^2^ = 68%). MBIs were associated with a reduction in the severity of depression in the short term (19 trials; 1874 participants; SMD, −0.73; 95% CI; −1.00 to −0.46; *I*^2^ = 86%) and the medium term (8 trials; 891 participants; SMD, −0.85; 95% CI, −1.35 to −0.35; *I*^2^ = 91%) and improved health-related quality of life in patients in the short term (9 trials; 1108 participants; SMD, 0.51; 95% CI, 0.20 to 0.82; *I*^2^ = 82%) and the medium term (5 trials; 771 participants; SMD, 0.29; 95% CI, 0.06 to 0.52; *I*^2^ = 57%).

**Conclusions and Relevance:**

In this study, MBIs were associated with reductions in anxiety and depression up to 6 months postintervention in adults with cancer. Future trials should explore the long-term association of mindfulness with anxiety and depression in adults with cancer and determine its efficacy in more diverse cancer populations using active controls.

## Key Points

**Question:**

Are mindfulness-based interventions associated with decreased anxiety in adults with cancer?

**Findings:**

In this systematic review and meta-analysis of 28 randomized clinical trials with 3053 participants, mindfulness-based interventions were associated with reductions in the severity of anxiety in adults with cancer up to 6 months after delivery of mindfulness sessions compared with usual care, waitlist control, or no intervention; a concomitant reduction in the severity of depression and improvement in health-related quality of life was also observed. None of the trials used mindfulness-based interventions in children with cancer.

**Meaning:**

Mindfulness-based interventions were associated with a reduction in anxiety and depression in adults with cancer.

## Teaser

This systematic review and meta-analysis of 28 randomized controlled trials examines the effectiveness in mindfulness-based interventions in reducing anxiety and depression in adult patients with cancer.

## Introduction

Anxiety is highly prevalent among patients with cancer.^1,2,3^ Approximately 10% to 19% of adults with cancer experience anxiety during or after cancer treatment.^2,3,4^ Similarly, 14% to 27% of children with cancer experience anxiety.^5,6^ Cancer-related anxiety is multifactorial and may stem from patients’ psychologic response to the diagnosis and treatment of cancer and from changes in body image, sexual function, work, and social interactions.^1,7^ Anxiety reduces patients’ health-related quality of life (HRQoL) and can also reduce treatment adherence.^8,9,10^ In 2014, the American Society of Clinical Oncology recommended individual and group psychologic interventions, such as cognitive-behavioral therapy, applied relaxation, and pharmacologic approaches, to treat cancer-related anxiety.^11^ Mindfulness, a type of mind and body intervention, was not identified as an intervention for the management of anxiety in these guidelines.^11,12^

Mindfulness is a technique whereby a person becomes purposefully cognizant of the present moment and learns to address their thoughts, feelings, and sensations in a nonjudgmental manner.^13,14^ Many patients with cancer use mindfulness to manage anxiety, depression, and emotional distress during and after their cancer treatment.^15,16,17,18,19^ Several randomized clinical trials (RCTs) conducted during the past decade^18,19,20,21,22^ have examined the effect of mindfulness-based interventions on anxiety, depression, psychological distress, and HRQoL in adults with cancer. The results of these trials are conflicting about the effect of mindfulness on anxiety.^18,20,21,23^

Previous systematic reviews have evaluated the associations of mindfulness-based stress reduction (MBSR), a widely used mindfulness-based intervention, with psychologic symptoms and HRQoL among patients with breast cancer.^24,25,26,27^ These reviews did not explore the association of mindfulness with anxiety in other cancer types and did not examine different types of mindfulness-based interventions, such as mindfulness-based cognitive therapy (MBCT) or mindfulness-based cancer recovery (MBCR).^24,25,26,27^ Since the publication of these systematic reviews, several RCTs have been performed. These trials evaluated the effect of various mindfulness-based interventions in patients with cancer.

In light of the growing literature on the use of mindfulness-based interventions for the reduction of anxiety and depression among patients with cancer, we conducted this systematic review and meta-analysis to synthesize the existing evidence. Our primary objective was to determine whether mindfulness-based interventions were associated with reductions in the severity of anxiety in adults and children with cancer. Our secondary objective was to determine whether these associations varied by cancer type, intervention, or methods of the study.

## Methods

### Search Strategy and Identification of Studies

Our systematic review was conducted according to an a priori protocol. We searched MEDLINE, Embase, Cochrane Central Register of Controlled Trials, CINAHL, PsycINFO, and SCOPUS from database inception to May 2019 to identify relevant citations of published RCTs, using individualized systematic search strategies for each database. The search strategy was developed by a health sciences librarian and peer reviewed by a second librarian using the Peer Review of Electronic Search Strategies checklist.^28^ The search strategy is presented in eTable 1 in the Supplement. We contacted study authors when required to request pertinent unpublished data or seek clarification on study methods or results. Reference lists of narrative and systematic reviews and of the included trials were hand-searched for additional citations. Our findings are reported according to the Preferred Reporting Items for Systematic Reviews and Meta-Analyses (PRISMA) reporting guideline.

### Study Selection and Data Extraction

We included RCTs comprised of a patient population of adults (≥18 years) or children (<18 years) with cancer or who were undergoing hematopoietic stem cell transplant as a treatment for cancer (≥80% of the study population). Enrolled patients were randomized to mindfulness-based interventions or usual care, waitlist, no intervention, or sham control groups for the management of anxiety. We excluded observational, quasi-randomized, crossover, or cluster-randomized trial designs and trials that did not report any outcomes of interest to this review. We did not restrict inclusion by language or date of publication.

Two reviewers (S.O. and J.Y.) independently evaluated the titles and abstracts. The full text of any citation considered potentially relevant by either reviewer was retrieved and assessed for eligibility. The inclusion of trials in this meta-analysis was determined by agreement of the 2 reviewers with discrepancies resolved by a third reviewer (L.S., A.M.A-S, or R.Z.).

We extracted data from included trials using standardized data extraction forms. Study-level variables included the year of publication and enrollment, country of study, age of participants (adults vs children), cancer diagnosis (breast, prostate, other cancer, or more than 1 cancer type), cancer stage (nonmetastatic, metastatic, or both), the timing of intervention (during cancer treatment, following completion of treatment, or both), selective enrollment of palliative care patients (author defined), presence of anxiety as an eligibility criterion for recruitment (author-defined threshold), characteristics of the intervention and control groups, and anxiety, depression, and HRQoL scores up to 1 month, between 1 and 6 months inclusive, and from 6 months up to 1 year postintervention. We decided a priori to include overall or global HRQoL scores for the data synthesis. General health HRQoL scores were extracted from the studies that did not provide global or overall HRQoL scores.

### Outcomes

Incorporating recommendations from patients and stakeholders, our primary outcome was self-reported short-term (≤1 month postintervention) severity of anxiety measured by any anxiety assessment scale. For studies that used more than 1 type of anxiety assessment scale, we defined a hierarchy of anxiety assessment scales based on the most prevalent scale used among the included trials to use in the primary analysis (eTable 2 in the Supplement). For instance, if a trial reported anxiety using both the Hospital Anxiety and Depression Scale–Anxiety (HADS-A) and the Beck Anxiety Inventory scale, the anxiety scores measured by the HADS-A scale were used for the primary outcome. Our secondary outcomes were medium-term (1 to ≤6 months postintervention) and long-term (>6 to 12 months postintervention) severity of self-reported anxiety, self-reported severity of depression across all the depression scales used in the included studies, patients’ overall or general HRQoL scores assessed by self-reported HRQoL questionnaires, and caregivers’ HRQoL scores assessed by self-reported questionnaires in short-, medium-, and long-term follow up.

### Intervention and Comparator

Mindfulness was defined as a technique in which a person learns to focus attention in the present to become mindful of thoughts, feelings, and sensations and observes them in a nonjudgmental way to achieve greater calmness, physical relaxation, and psychologic balance.^13,14^ For our review, we included the mindfulness-based interventions (eg, MBSR, MBCT, MBCR, or mindfulness-based art therapy [MBAT]) that use mindfulness as the foundation of a therapeutic intervention instead of as an ancillary component.^13,14^ Interventions that include mindfulness as 1 of many elements (eg, yoga, Qigong, and tai chi) were not included because it is difficult to determine the effect of mindfulness in the setting of the physical movement. The control groups in the trials were usual care, waitlist, no intervention, or sham control.

### Risk of Bias Assessment

We assessed the internal validity of included trials using the Cochrane Collaboration Risk of Bias tool.^28,29^ This tool categorizes the overall risk of study bias and consists of 6 domains (ie, sequence generation, allocation concealment, blinding, incomplete outcome data, selective outcome reporting, and other sources of bias). Each domain is rated low risk, unclear risk, or high risk. Information regarding the risk of bias was used to guide sensitivity analyses and explore sources of heterogeneity. Discrepancies in assessments between the 2 reviewers were resolved by consensus or by a third reviewer (A.M.A-S. or L.S.), as required.

### Statistical Analysis

We used the standardized mean difference (SMD) to synthesize data across studies, which measured anxiety, depression, or HRQoL using a variety of scales.^29^ Weighted mean difference (WMD) was used to synthesize data for analyses concerning specific anxiety and depression scales. A summary effect estimate less than 0 indicates that the mean score was lower (ie, better) in the mindfulness group compared with the control group. The generic inverse variance method was used to weight the effects. A random-effects model was used for all analyses. We assessed statistical heterogeneity between trials using the *I*^2^ statistic.^30^

For the primary outcome, we evaluated potential publication bias using funnel plot analysis and visual inspection of the forest plot.^31^ In the event of potential publication bias, the trim-and-fill technique was used to determine the effect of potential bias using Comprehensive Meta-analysis software, version 2 (Biostat).^32^ Meta-analyses were conducted using Review Manager, version 5.3.5 (The Cochrane Collaboration). All tests of statistical inference reflect a 2-sided α < .05.

We evaluated the following factors in subgroup analyses: (1) type of cancer (ie, breast cancer vs mixed cancer population and other cancer types), (2) cancer stage (ie, nonmetastatic vs metastatic vs both nonmetastatic and metastatic), (3) timing of intervention (ie, during cancer treatment vs after cancer treatment vs both during and after cancer treatment), (4) mindfulness-based intervention type (ie, MBCT vs MBSR vs MBCR vs MBAT), (5) group vs nongroup or individual delivery setting, (6) number of weeks of intervention (<8 weeks vs ≥8 weeks), (7) sequence generation (ie, low vs high or unclear risk of bias), and (8) allocation concealment (low vs unclear or high risk of bias). We determined whether the mindfulness-based intervention effect varied by subgroups using the *P* value for interaction.

## Results

**Figure 1.**
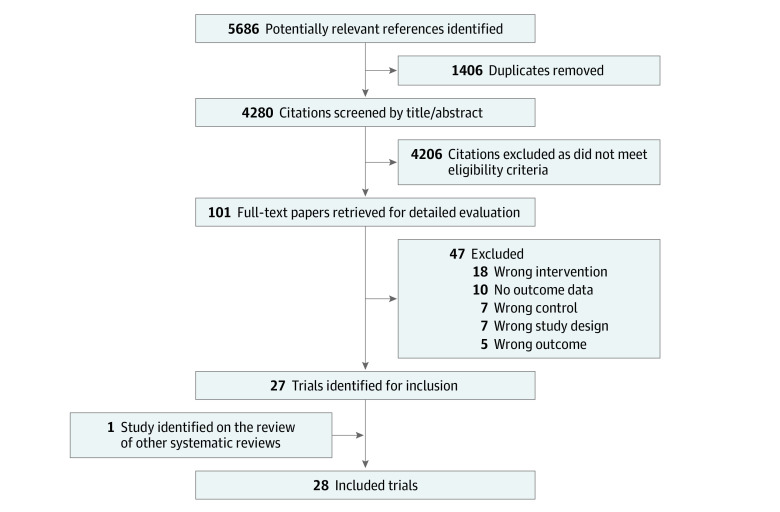
Flow Diagram of Trial Identification and Selection

Our electronic search strategy identified 5686 citations, of which 27 trials met the eligibility criteria (Figure 1).^16,18,19,20,21,22,33,34,35,36,37,38,39,40,41,42,43,44,45,46,47,48,49,50,51,52,53,54^ We included 1 additional trial identified through hand searching. One trial by Compen et al^21^ randomized patients to in-person MBCT, internet-based MBCT, and usual care cohorts and thus represented 2 separate analyses, for an overall total of 29 mindfulness-based comparisons (3053 participants). All trials were published as full-text articles in English. These trials were conducted in 13 countries from 4 continents (Table 1). None of the trials included children or adolescents with cancer. Trials of breast cancer patients were the most common (12 trials [42.8%]). Even in trials including more than 1 type of cancer (11 trials), breast cancer was the most common diagnosis in all but 1.^21,40,44,49,51,53^

**Table 1.** Characteristics of 28 Studies Included in the Systematic Review

Source; Country^a^	Age range, y	Cancer diagnosis	Cancer stage	Timing	Total patients, No.	Intervention			
						Type	Delivery	Mode	Duration, wk
Liu et al,^33^ 2019; China	NA	Thyroid	Nonmetastatic	On therapy	120	MBSR	Group	In-person	8
Franco et al,^34^ 2019; Spain	NA	Breast	NA	NA	36	Mindfulness-based program	Group	In-person	7
Compen et al,^21^ 2018; the Netherlands	NA	>1	Nonmetastatic and metastatic	Both	116	MBCT and eMBCT	Group	In-person and web-based	8
Lorca et al,^35^ 2018; Spain	NA	>1	NA	Both	108	Mindfulness-based program	Individual	CD	50-60 min
Russell et al,^36^ 2018; Australia	22-78	Melanoma	Nonmetastatic	Off therapy	69	Mindfulness-based program	Nongroup	Web-based	6
Chambers et al,^37^ 2017; Australia and New Zealand	NA	Prostate	Nonmetastatic and metastatic	Both	189	MBCT	Group	In-person	8
Kenne et al,^38^ 2017; Sweden	34-80	Breast	NA	Off therapy	120	MBSR	Group	In-person	8
Schellekens et al,^39^ 2017; the Netherlands	NA	Lung	Nonmetastatic and metastatic	Both	63	MBSR	Group	In-person	8
Zhang et al,^22^ 2017; China	17-71	Leukemia	NA	On therapy	65	Mindfulness-based psychological care	Nongroup	In-person	5
Zhang et al,^19^ 2017; China	30-62	Breast	Nonmetastatic	Off therapy	60	MBSR	Group	In-person	8
Blaes et al,^40^ 2016; United States	36-79	>1	Nonmetastatic	Off therapy	42	MBCR	Group	In-person	8
Jang et al,^41^ 2016; Korea	NA	Breast	Nonmetastatic	Off therapy	24	MBAT	Group	In-person	12
Johannsen et al,^42^ 2016; Denmark	NA	Breast	Nonmetastatic	Off therapy	129	MBCT	Group	In-person	8
Lengacher et al,^18^ 2016; United States	NA	Breast	Nonmetastatic	Off therapy	322	MBSR	Group	In-person	6
Vaziri et al,^20^ 2016; Iran	NA	Breast	Nonmetastatic	On therapy	20	MBCT	Group	NA	8
Bower et al,^43^ 2015; United States	28.4-60	Breast	Nonmetastatic	Off therapy	71	Mindful awareness practices	Group	In-person	6
Johns et al,^44^ 2015; United States	NA	>1	Nonmetastatic and metastatic	Off therapy	35	MBSR	Group	In-person	7
Kingston et al,^45^ 2015; Ireland	NA	>1	NA	Both	16	MBCT	Group	In-person	8
Zernicke et al,^46^ 2014; Canada	29-79	>1	Nonmetastatic and metastatic	Off therapy	62	MBCR	Group	Web-based	8
Würtzen et al,^47^ 2013; Denmark	NA	Breast	Nonmetastatic	Both	336	MBSR	Group	In-person	8
Hoffman et al,^16^ 2012; United Kingdom	18-80	Breast	Nonmetastatic	Off therapy	229	MBSR	Group	In-person	8
Henderson et al,^48^ 2013; United States	NA	Breast	Nonmetastatic	Both	111	MBSR	Group	In-person	20
Lerman et al,^49^ 2012; United States	NA	>1	NA	Both	77	MBSR	Group	In-person	8
Bränström et al,^50^ 2010; Sweden	NA	>1	NA	NA	85	MBSR	Group	In-person	8
Foley et al,^51^ 2010; Australia	24-78	>1	Nonmetastatic and metastatic	NA	115	MBCT	Group	In-person	8
Lengacher et al,^52^ 2009; United States	NA	Breast	Nonmetastatic	Off therapy	84	MBSR	Group	In-person	6
Monti et al,^53^ 2006; United States	26-82	>1	Nonmetastatic and metastatic	Both	111	MBAT	Group	In-person	8
Speca et al,^54^2000; Canada	NA	>1	Nonmetastatic and metastatic	Both	109	MBSR	Group	In-person	7

Abbreviations: eMBCT, web-based mindfulness-based cognitive therapy; MBAT, mindfulness-based art therapy; MBCR, mindfulness-based cancer recovery; MBSR, mindfulness-based stress reduction; NA, not available.

^a^ All included trials were conducted among adults.

The 2 most common mindfulness-based interventions used in the trials were MBSR (13 trials [46.4%]) and MBCT (6 trials [24.1%]). Trials used usual care (14 trials) and waitlist (14 trials) equally as comparators. The median duration of intervention was 8 weeks (range, 1 hour to 20 weeks). Anxiety assessment scales used across all included trials are listed in eTable 2 in the Supplement. Among the 12 reported anxiety scales, the 2 most common scales were the HADS-A (5 trials) and the State-Trait Anxiety Inventory (STAI; 5 trials).

The risk of bias assessment is presented in eFigure 1 in the Supplement. As participants and outcome assessors could not be blinded because of the nature of the interventions and patient-reported anxiety outcome, all trials were considered at a high risk of performance and detection bias.

### Primary Outcome

**Figure 2.**
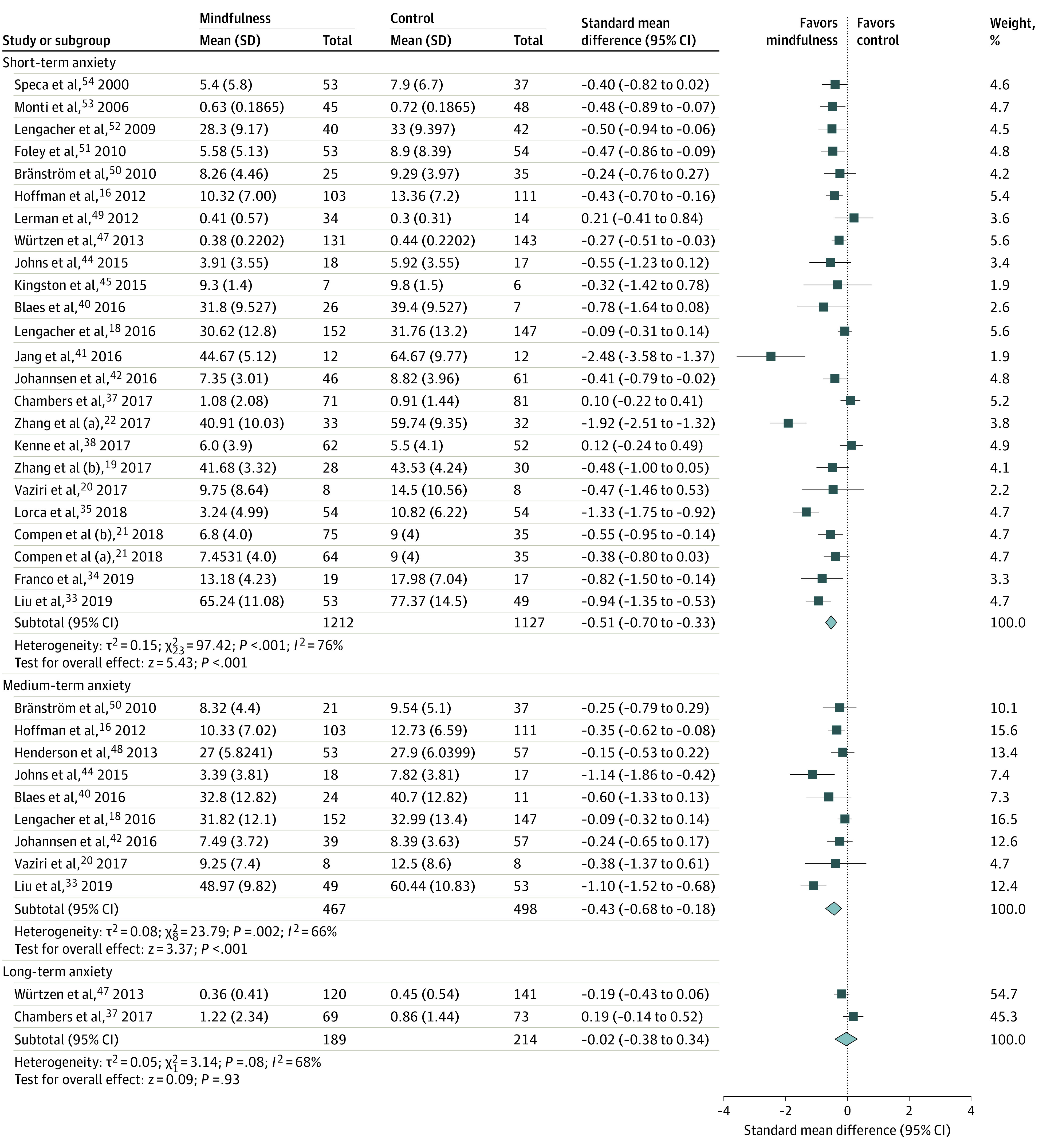
Forest Plot of Association of Mindfulness-Based Interventions With Anxiety in the Short Term, Medium Term, and Long Term The size of the square reflects study’s relative weight, and the diamond represents the aggregate standardized mean difference and 95% CI. Studies that have multiple listed entries perform multiple analyses between distinct patient groups.

Mindfulness-based interventions were associated with reductions in the severity of short-term anxiety (23 trials; 2339 participants; SMD, −0.51; 95% CI, −0.70 to −0.33; *I*^2^ = 76%) (Figure 2). Similar results were found when limiting the analysis to trials that measured anxiety using the HADS-A (6 trials; 503 participants; WMD, −1.03; 95% CI, −1.82 to −0.23; *I*^2^ = 32%) or STAI scales (5 trials; 580 participants; WMD, −4.00; 95% CI, −5.21 to −2.78; *I*^2^ = 80%). On sensitivity analysis, the effect estimate was reduced (22 trials; 2231 participants; SMD, −0.46; 95% CI, −0.64 to −0.29; *I*^2^ = 72%) when the trial by Lorca et al^35^ was excluded because of the very short duration of the intervention (50-60 minutes). Examination of the funnel plot for the primary outcome showed the potential absence of small-size studies showing no effect of mindfulness; however, no statistically significant evidence of publication bias was present using trim-and-fill methods (eFigure 2 in the Supplement).

Table 2 illustrates the a priori subgroup analyses performed to explore heterogeneity in the association of mindfulness with a reduction in short-term anxiety. Subgroup analyses that included the type and stage of cancer, timing of treatment, intervention type, duration of treatment, delivery setting, number of weeks of intervention, and the presence of adequate randomization or allocation concealment could not explain the heterogeneity in the effect of mindfulness (Table 2).

**Table 2.** Association of Mindfulness-Based Interventions With Short-Term Anxiety by Patient, Intervention, and Methodological Factors in 28 Included Studies

Subgroup	Trials, No.	Patients, No.	SMD (95% CI)	*I*^2^, %	*P* value for interaction
Type of cancer					
Breast	10	1224	−0.40 (−0.62 to −0.17)	68	.33
>1 Cancer and other cancer types	14	1115	−0.57 (−0.86 to −0.29)	76	
Cancer stage					
Nonmetastatic	10	1209	−0.52 (−0.73 to −0.31)	69	.31
Metastatic	0	0	NA		
Metastatic and nonmetastatic	7	686	−0.36 (−0.55 to −0.16)	37	
Timing of intervention					
During cancer treatment	3	183	−1.16 (−1.94 to −0.38)	78	.19
After cancer treatment	9	966	−0.43 (−0.70 to −0.17)	71	
Both during and after cancer treatment	9	989	−0.39 (−0.68 to −0.11)	76	
Intervention type					
Mindfulness-based stress reduction	11	1376	−0.32 (−0.49 to −0.14)	57	.52
Mindfulness-based cognitive therapy	7	604	−0.32 (−0.53 to −0.12)	31	
Mindfulness-based art therapy	2	117	−1.41 (−3.36 to 0.54)	91	
Mindfulness-based cancer recovery	1	33	−0.78 (−1.64 to 0.08)	NA^a^	
Delivery setting					
Group	21	2067	−0.39 (−0.54 to −0.24)	60	.07
Nongroup	3	272	−1.19 (−2.05 to −0.34)	90	
Length of intervention, wks					
<8	7	715	−0.78 (−1.27 to −0.29)	88	.14
≥8	17	1624	−0.39 (−0.57 to −0.21)	71	
Adequate sequence generation					
Yes	18	2028	−0.43 (−0.61 to −0.26)	71	.23
Unclear	6	311	−0.83 (−1.46 to −0.21)	82	
Adequate allocation concealment					
Yes	10	1361	−0.36 (−0.54 to −0.17)	63	.32
Unclear	14	978	−0.68 (−1.02 to −0.34)	76	

Abbreviations: NA, not applicable; SMD, standardized mean difference.

^a^ *I*^2^ is not calculated here because only 1 study reported this outcome.

### Secondary Outcomes

Mindfulness-based interventions reduced the severity of medium-term (1 to 6 months postintervention) anxiety (9 trials; 965 participants; SMD, −0.43; 95% CI, −0.68 to −0.18; *I*^2^ = 66%) (Figure 2). Mindfulness-based interventions were not associated with a long-term (>6 months to 1 year postintervention) reduction in anxiety (2 trials; 403 participants; SMD, −0.02; 95% CI, −0.38 to 0.34; *I*^2^ = 68%) (Figure 2).

**Figure 3.**
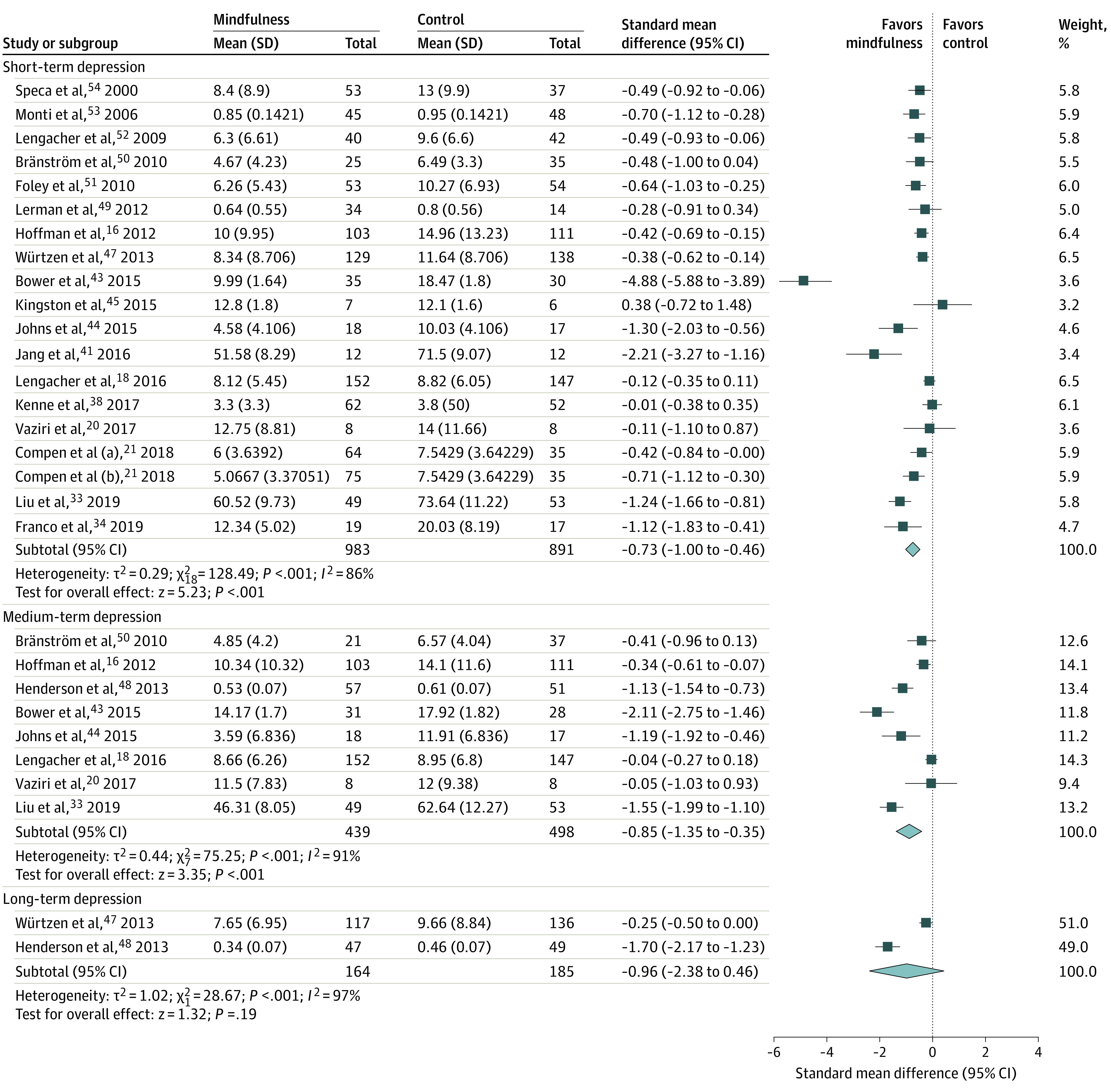
Forest Plot of Association of Mindfulness-Based Interventions With Depression in Short Term, Medium Term, and Long Term The size of the square reflects study’s relative weight, and the diamond represents the aggregate standardized mean difference and 95% CI. Studies that have multiple listed entries perform multiple analyses between distinct patient groups.

Mindfulness-based interventions were associated with reductions in the severity of short-term depression (19 trials; 1874 participants; SMD, −0.73; 95% CI, −1.00 to −0.46; *I*^2^ = 86%) (Figure 3) and medium-term depression (8 trials; 891 participants; SMD, −0.85; 95% CI, −1.35 to −0.35; *I*^2^ = 91%) (Figure 3). The list of all depression assessment scales used in the included trials is provided in eTable 3 in the Supplement. Mindfulness-based interventions were associated with a reduction in depression when the analysis was limited to trials reporting the HADS-D (5 trials; 396 participants; WMD, −1.16; 95% CI, −1.94 to −0.38; *I*^2^ = 54%). Mindfulness was not associated with a reduction in the severity of depression in the long term (2 trials; 349 participants; SMD, −0.96; 95% CI, −2.38 to 0.46; *I*^2^ = 97%) (Figure 3).

Mindfulness-based interventions were associated with improved overall HRQoL scores in the short term (9 trials; 1108 participants; SMD, 0.51; 95% CI, 0.20 to 0.82; *I*^2^ = 82%) and medium term (5 trials; 771 participants; SMD, 0.29; 95% CI, 0.06 to 0.52; *I*^2^ = 57%). The included HRQoL assessment scales are listed in eTable 4 in the Supplement. The only trial reporting the effect of mindfulness on long-term HRQoL did not demonstrate any improvement in HRQoL (1 trial; 153 participants; WMD, 0.78; 95% CI, −5.98 to 7.54). None of the included trials reported caregivers’ HRQoL.

## Discussion

In this systematic review and meta-analysis, we found that mindfulness-based interventions were associated with reductions in the severity of anxiety in the short term and medium term, but not in the long term, among adults with cancer. Mindfulness was associated with a significant improvement in short-term and medium-term, but not in long-term, depression or HRQoL.

Our study builds on previously published systematic reviews.^24,25,26,27^ We incorporated diverse mindfulness-based interventions across all cancer populations, whereas the majority of the published systematic reviews were narrowly focused on MBSR interventions in patients with breast cancer.^24,26,27^ The only published systematic review examining various types of mindfulness interventions across different cancer diagnoses in adults included only 9 trials and did not use rigorous methods for meta-analysis and assessing the quality of included trials.^55^ In contrast, we used robust methods for identifying trials and synthesizing data. Our review identified a larger number of trials, synthesized the effect of treatment at various time points, and assessed subgroup differences and the risk of bias across included studies. The larger sample size improved the precision of the effect estimates and enabled us to perform subgroup analysis to explore heterogeneity.

An SMD of 0.51 suggests a moderate reduction in short-term anxiety associated with mindfulness.^56,57^ This association with reduction in short-term anxiety remained significant even when studies using the HADS-A and STAI were analyzed separately. The minimum clinically important difference (MCID) associated with HADS-A and STAI has not been identified for patients with cancer in the literature. The reported MCID for HADS-A among patients with chronic obstructive pulmonary disease is 1.5.^58^ If we extrapolate the MCID of the HADS-A scale from patients with chronic obstructive pulmonary disease to patients with cancer, the observed reduction in short-term anxiety on the HADS-A scale (1.03) is lower than the clinically meaningful threshold. Similar to anxiety, the observed short-term association of mindfulness with depression could not reach a clinically significant threshold. However, it should be noted that behavioral therapies, recommended by American Society of Clinical Oncology as 1 of the modalities for the treatment of anxiety and depression in adults with cancer, are associated with small to moderate effect sizes, similar to mindfulness-based interventions.^11,59^ Furthermore, mindfulness-based interventions also positively affect other symptoms, such as fatigue, distress, and pain in patients with cancer.^60,61^ Therefore, we suggest that mindfulness-based interventions should be considered an effective treatment option for reducing short-term and medium-term anxiety among adults with cancer. Comparing mindfulness-based interventions with other effective interventions for anxiety, such as drugs and behavioral therapies, should be the focus of future research in adults with cancer.

We also demonstrated that, in the longer term, mindfulness-based interventions were associated with little or no benefit on anxiety, depression, or quality of life. Our confidence in the long-term effects of mindfulness-based interventions is low because only 2 trials reported the long-term effects of mindfulness. It is plausible that the absence of booster or follow-up sessions after an initial 8 to 12 weeks of intervention might have diminished the durability of effect. As mindfulness is a skill-building approach, booster sessions may provide participants with the opportunity to master difficult skills and to generalize the mindfulness skills to newly encountered situations in life.

The effect of mindfulness on short-term anxiety did not differ by the assessed patient, intervention, and study characteristics. Specifically, the short-term benefits of mindfulness on anxiety were dependent neither on the type of cancer nor on the treatment phase. These results suggest that examined mindfulness-based interventions could result in the reduction of short-term anxiety of adults regardless of cancer type or stage of treatment. Furthermore, the association of mindfulness with reductions in short-term anxiety was not restricted to studies providing 8 weeks or more of mindfulness and group sessions; instead, the reduction in anxiety was marginally higher when mindfulness was delivered in the individual setting. In clinical practice, mindfulness-based interventions are typically offered in a group setting during an 8-week period.^21,26,37^ The longer-duration group sessions can be challenging for patients who either have complications related to cancer or are on active treatment. Young adults with cancer who are well connected with technology may also prefer shorter mindfulness sessions delivered by telehealth, video-interface, or a mobile application.^62^ Our findings suggest that individually delivered or shorter duration mindfulness-based interventions can be offered to the patients with cancer who cannot commit to group sessions or 8 weeks of intervention due to their illnesses or other barriers.

### Limitations

Our systematic review has limitations that deserve comment. We cannot be certain that the observed treatment gains were exclusively due to the mindfulness intervention and not because of the facilitator’s attention, group support, and social interaction, given that none of the trials included an attention control group that matched the intervention group on all of these elements. These nonspecific factors are inherent components of the mindfulness intervention and can potentially affect the outcome. Women were overrepresented in the trials with mixed cancer populations,^21,49,51,53^ and 43% of included trials were done in patients with breast cancer who had completed curative treatment. The effect size for men in the gender-inclusive trials was not reported separately.^21,49,51,53^ The only trial that exclusively enrolled men with advanced prostate cancer did not show any reduction in anxiety.^37^ Men may be less receptive and responsive to mindfulness because of their different coping mechanisms than women.^63^ Therefore, further investigation of how gender affects the efficacy of mindfulness is warranted. There was also no representation of patients in palliative care or undergoing hematopoietic stem cell transplant in the included trials. None of the included trials were conducted in children or in patients with metastatic disease exclusively. Consequently, the results of this meta-analysis may not be generalizable to all cancer populations and settings. Most included trials did not use standardized tools to measure the total time spent practicing mindfulness per week or reported changes in the mindfulness. These factors may explain the heterogeneity in the measured effect, which subgroup analysis did not demonstrate. Other unmeasured patient characteristics in the included trials may also have contributed to the heterogeneity. Finally, imprecision, related to the small sample size, limited our confidence in the long-term effects of mindfulness-based interventions on the studied outcomes.

## Conclusions

In this study, mindfulness-based interventions were associated with short-term and medium-term reductions in anxiety and depression along with improved quality of life among adult patients with cancer. Future studies should assess the efficacy of mindfulness in different settings, include diverse cancer populations such as children and men, and compare mindfulness with other effective interventions for anxiety and depression.

## Notes

Supplement. **eTable 1.** Search Strategy **eTable 2.** Self-reported Anxiety Assessment Scales Used in the Included Trials (n = 28) **eTable 3.** Self-reported Depression Assessment Scales Used in the Included Trials (n = 28) **eTable 4.** Self-reported Health-related Quality of Life Assessment Scales Used in the Included Trials (n = 28) **eFigure 1.** Summary of the Risk of Bias of Studies Included in the Systematic Review (n = 28) **eFigure 2.** Funnel Plot “Trim-and-Fill” Technique Assessing Publication Bias for Short-term Reduction in Severity of Anxiety
