# Supplementary material for: Association of Mindfulness-Based Interventions With Anxiety Severity in Adults With Cancer: A Systematic Review and Meta-analysis
Source: JAMA Netw Open. 2020 Aug 7;3(8):e2012598. doi: 10.1001/jamanetworkopen.2020.12598 (PMC7414391; doi:10.1001/jamanetworkopen.2020.12598)
Supplement: Supplement. — eTable 1. Search Strategy eTable 2. Self-reported Anxiety Assessment Scales Used in the Included Trials (n = 28) eTable 3. Self-reported Depression Assessment Scales Used in the Included Trials (n = 28) eTable 4. Self-reported Health-related Quality of Life Assessment Scales Used in the Included Trials (n = 28) eFigure 1. Summary of the Risk of Bias of Studies Included in the Systematic Review (n = 28) eFigure 2. Funnel Plot “Trim-and-Fill” Technique Assessing Publication Bias for Short-term Reduction in Severity of Anxiety [file jamanetwopen-3-e2012598-s001.pdf]

Supplementary Online Content

Oberoi S, Yang J, Woodgate RL, et al. Association of mindfulness-based interventions with anxiety severity in adults with cancer: a systematic review and meta-analysis. *JAMA Netw Open*. 2020;3(8):e2012598. doi:10.1001/jamanetworkopen.2020.12598

eTable 1. Search Strategy

eTable 2. Self-reported Anxiety Assessment Scales Used in the Included Trials (n=28)

eTable 3. Self-reported Depression Assessment Scales Used in the Included Trials (n=28)

eTable 4. Self-reported Health-related Quality of Life Assessment Scales Used in the Included Trials (n=28)

eFigure 1. Summary of the Risk of Bias of Studies Included in the Systematic Review (n= 28)

eFigure 2. Funnel Plot "Trim and Fill" Technique Assessing Publication Bias for Short-term Reduction in Severity of Anxiety

This supplementary material has been provided by the authors to give readers additional information about their work.

© 2020 Oberoi S et al. *JAMA Netw Open.*

eTable 1. Search strategy[a]

**Ovid**	
exp neoplasms/	3129461
exp hematopoietic stem cell transplantation/ or stem cell transplantation/ or hematopoietic stem cell mobilization/	64127
(cancer* or neoplas* or tumo?r* or malignan* or metasta* or oncogen* or oncolog* or hepatoma* or hepatoblastoma* or hepato-blastoma* or carcinoma* or adenoma? or adenocarcinoma? or adeno-carcinoma? or blastoma? or carcinosarcoma? or carcino-sarcoma? or leukemia? or leukaemia? or lymphoma? or melanoma? or mesenchymoma? or mesothelioma? or sarcoma? or thymoma?).tw,kf	3477981
(HSCT or ((h?ematopoietic or h?emato-poietic) adj3 (transplant* or sct or bct or mobili#ation)) or pbsct or pbct or psct or ((peripheral or pbsc or pbscs) adj3 transplant*) or autohct or autopbsct or autopbsc or autopsc or cbsct or ((autologous or auto-logous or auto or allogeneic or allo-geneic or homologous or homo-logous) adj hct)).tw,kf	32738
or/1-4	4171187
exp Anxiety/ or exp fear/	102154
exp Anxiety Disorders/ or stress disorders, traumatic/ or psychological trauma/ or stress disorders, post-traumatic/ or stress disorders, traumatic, acute/ or exp phobic disorders/	103858
(anxiet* or stress disorder* or psychological trauma* or ptsd or ptsds or moral injur* or post trauma* or posttrauma* or (stress* adj2 trauma*)).tw,kf	230041
(anxious* or fear or fears or fearful* or nervous* or angst or apprehensi* or panic* or phobic or phobia*).tw,kf	402553
or/6-9	658915
exp mind-body therapies/ or exp musculoskeletal manipulations/ or exp exercise movement techniques/ or exp spiritual therapies/ or exp acupuncture therapy/	89741
((mind? adj1 body) or acupunctur* or acupotom* or electroacupunctur* or electro-acupunctur* or moxibustion or moxabustion or shaman* or witch* or sorcer* or radiesthesi* or aroma therap* or aromatherap* or (essential adj2 oil*) or bio-feedback* or myo-feedback* or psycho-physiologic feedback* or neuro-feedback* or biofeedback* or myofeedback* or psychophysiologic feedback* or neurofeedback* or alpha feedback* or brainwave feedback* or qigong or qi gong or ch'i kung or ((breath* or coping or respiratory) adj2 (exercise* or technique* or train*)) or relax* or hypnos#s or hypnotherap* or hypno-therap* or hypnotism or autogenic training or self-hypno* or imagery or reverie or laughter or laughing or meditat* or mindful* or psychodrama* or psycho-drama* or tai chi or tai ji or taiji or therapeutic touch* or healing touch* or reiki or yoga or energy therap* or ((musculoskeletal* or musculo-skeletal* or orthop?edic* or osteopathic* or spinal* or cervical* or lumbar*) adj2 manipulat*) or ((manipulat* or manual) adj2 therap*) or chiropractic* or soft tissue therap* or acupressure* or chih ya or shiatsu or shiatzu or zhi ya or bodywork* or massag* or rolfing or zone therap* or reflex therap* or reflexolog* or reflexotherap* or reflexo-therap* or manual lymph* or ((spiritual or faith or mental* or religion* or religious*) adj3 (healing or heal or healed or heals)) or prayer* or magic* or body align* or pilates or movement technique* or alexander technique* or feldenkrais or structural integration* or trager approach* or psycho-physical integration* or psychophysical integration* or mentastic* or ((art or music) adj3 therap*) or dance or dancing).tw,kf	302728
or/11-12	342288
randomized controlled trial.pt	475636
controlled clinical trial.pt	92894
Clinical Trials as topic/	185927
((singl* or doubl* or trebl* or tripl*) adj (blind* or dumm* or mask*)).tw,kf	161803
PLACEBOS/	34216
(placebo* or sham).tw,kf	278392

© 2020 Oberoi S et al. *JAMA Netw Open.*

(randomized or randomised or randomly or rct).tw,kf	804608
trial.ti	193712
or/14-21	1311132
5 and 10 and 13 and 22	590
exp animals/ not humans/	4543138
23 not 24	587
**CENTRAL**	
[mh neoplasms]	67536
[mh "hematopoietic stem cell transplantation"] or [mh ^"stem cell transplantation"] or [mh ^"hematopoietic stem cell mobilization"]	1730
(cancer* or neoplas* or tumo?r* or malignan* or metasta* or oncogen* or oncolog* or hepatoma* or hepatoblastoma* or hepato-blastoma* or carcinoma* or adenoma* or adenocarcinoma* or adeno-carcinoma* or blastoma* or carcinosarcoma* or carcino-sarcoma* or leukemia* or leukaemia* or lymphoma* or melanoma* or mesenchymoma* or mesothelioma* or sarcoma* or thymoma*):ti,ab,kw	113392
(HSCT or ((h?ematopoietic or h?emato-poietic) NEAR/3 (transplant* or sct or bct or mobili#ation)) or pbsct or pbct or psct or ((peripheral or pbsc or pbscs) NEAR/3 transplant*) or autohct or autopbsct or autopbsc or autopsc or cbsct or ((autologous or auto-logous or auto or allogeneic or allo-geneic or homologous or homo-logous) NEXT hct)):ti,ab,kw	3235
{OR #1-#4}	138851
[mh Anxiety] or [mh fear]	7972
[mh "Anxiety Disorders"] or [mh ^"stress disorders, traumatic"] or [mh ^"psychological trauma"] or [mh ^"stress disorders, post-traumatic"] or [mh ^"stress disorders, traumatic, acute"] or [mh "phobic disorders"]	7925
(anxiet* or "psychological trauma" or ptsd or ptsds or (moral NEXT injur*) or (post NEXT trauma*) or posttrauma* or (stress* NEAR/2 (trauma* or disorder*))):ti,ab,kw	38931
(anxious* or fear or fears or fearful* or nervous* or angst or apprehensi* or panic* or phobic or phobia*):ti,ab,kw	29413
{OR #6-#9}	62595
[mh "mind-body therapies"] or [mh "musculoskeletal manipulations"] or [mh "exercise movement techniques"] or [mh "spiritual therapies"] or [mh "acupuncture therapy"]	11880
((mind? NEAR/1 body) or acupunctur* or acupotom* or electroacupunctur* or electro-acupunctur* or moxibustion or moxabustion or shaman* or witch* or sorcer* or radiesthesi* or (aroma NEXT therap*) or aromatherap* or (essential NEAR/2 oil*) or bio-feedback* or myo-feedback* or (psycho-physiologic NEXT feedback*) or neuro-feedback* or biofeedback* or myofeedback* or (psychophysiologic NEXT feedback*) or neurofeedback* or (alpha NEXT feedback*) or (brainwave NEXT feedback*) or qigong or "qi gong" or "ch'i kung" or ((breath* or coping or respiratory) NEAR/2 (exercise* or technique* or train*)) or relax* or hypnosis or hypnotherap* or hypno-therap* or hypnotism or "autogenic training" or self-hypno* or imagery or reverie or laughter or laughing or meditat* or mindful* or psychodrama* or psycho-drama* or "tai chi" or "tai ji" or taiji or (therapeutic NEXT touch*) or (healing NEXT touch*) or reiki or yoga or (energy NEXT therap*) or ((musculoskeletal* or musculo-skeletal* or orthopaedic* or orthopedic* or osteopathic* or spinal* or cervical* or lumbar*) NEAR/2 manipulat*) or ((manipulat* or manual) NEAR/2 therap*) or chiropractic* or "soft tissue therapy" or acupressure* or "chih ya" or shiatsu or shiatzu or "zhi ya" or bodywork* or massag* or rolfing or (zone NEXT therap*) or (reflex NEXT therap*) or reflexolog* or reflexotherap* or reflexo-therap* or (manual NEXT lymph*) or ((spiritual or faith or mental* or religion* or religious*) NEAR/3 (healing or heal or healed or heals)) or prayer* or magic* or (body NEXT align*) or pilates or (movement NEXT technique*) or (alexander NEXT technique*) or feldenkrais or (structural NEXT integration*) or "trager approach" or (psycho-physical NEXT integration*) or (psychophysical NEXT integration*) or mentastic* or ((art or music) NEAR/3 therap*) or dance or dancing):ti,ab,kw	46441
{OR #11-#12}	47060

© 2020 Oberoi S et al. *JAMA Netw Open.*

#5 and #10 and #13	605
**PsycINFO**	
exp neoplasms/	48144
stem cells/	3963
(cancer* or neoplas* or tumo?r* or malignan* or metasta* or oncogen* or oncolog* or hepatoma* or hepatoblastoma* or hepato-blastoma* or carcinoma* or adenoma? or adenocarcinoma? or adeno-carcinoma? or blastoma? or carcinosarcoma? or carcino-sarcoma? or leukemia? or leukaemia? or lymphoma? or melanoma? or mesenchymoma? or mesothelioma? or sarcoma? or thymoma?).tw	80135
(HSCT or ((h?ematopoietic or h?emato-poietic) adj3 (transplant* or sct or bct or mobili#ation)) or pbsct or pbct or psct or ((peripheral or pbsc or pbscs) adj3 transplant*) or autohct or autopbsct or autopbsc or autopsc or cbsct or ((autologous or auto-logous or auto or allogeneic or allo-geneic or homologous or homo-logous) adj hct)).tw	569
or/1-4	84879
exp Anxiety/ or exp fear/ or anxiety management/	82341
exp Anxiety Disorders/	78557
(anxiet* or stress disorder* or psychological trauma* or ptsd or ptsds or moral injur* or post trauma* or posttrauma* or (stress* adj2 trauma*)).tw	230399
(anxious* or fear or fears or fearful* or nervous* or angst or apprehensi* or panic* or phobic or phobia*).tw	189885
or/6-9	385720
mind body therapy/ or movement therapy/ or acupuncture/ or aromatherapy/ or exp biofeedback/ or exp relaxation therapy/ or autogenic training/ or witchcraft/ or exp religious practices/ or shamanism/ or mindfulness/ or hypnosis/ or exp hypnotherapy/ or autohypnosis/ or guided imagery/ or laughter/ or exp humor/ or psychodrama/ or massage/ or exp creative arts therapy/	60894
((mind? adj1 body) or acupunctur* or acupotom* or electroacupunctur* or electro-acupunctur* or moxibustion or moxabustion or shaman* or witch* or sorcer* or radiesthesi* or aroma therap* or aromatherap* or (essential adj2 oil*) or bio-feedback* or myo-feedback* or psycho-physiologic feedback* or neuro-feedback* or biofeedback* or myofeedback* or psychophysiologic feedback* or neurofeedback* or alpha feedback* or brainwave feedback* or qigong or qi gong or ch'i kung or ((breath* or coping or respiratory) adj2 (exercise* or technique* or train*)) or relax* or hypnos#s or hypnotherap* or hypno-therap* or hypnotism or autogenic training or self-hypno* or imagery or reverie or laughter or laughing or meditat* or mindful* or psychodrama* or psycho-drama* or tai chi or tai ji or taiji or therapeutic touch* or healing touch* or reiki or yoga or energy therap* or ((musculoskeletal* or musculo-skeletal* or orthop?edic* or osteopathic* or spinal* or cervical* or lumbar*) adj2 manipulat*) or ((manipulat* or manual) adj2 therap*) or chiropractic* or soft tissue therap* or acupressure* or chih ya or shiatsu or shiatzu or zhi ya or bodywork* or massag* or rolfing or zone therap* or reflex therap* or reflexolog* or reflexotherap* or reflexo-therap* or manual lymph* or ((spiritual or faith or mental* or religion* or religious*) adj3 (healing or heal or healed or heals)) or prayer* or magic* or body align* or pilates or movement technique* or alexander technique* or feldenkrais or structural integration* or trager approach* or psycho-physical integration* or psychophysical integration* or mentastic* or ((art or music) adj3 therap*) or dance or dancing).tw	123120
or/11-12	138106
(0300).md	22437
clinical trials/ or between groups design/	11346
((singl* or doubl* or trebl* or tripl*) adj (blind* or dumm* or mask*)).tw	24796
PLACEBO/	5203
(placebo* or sham).tw	49768
(randomized or randomised or randomly or rct).tw	134417
trial.ti	27289

or/14-20	184438
5 and 10 and 13 and 21	208
(exp animals/ or animal.po) not human.po	351310
22 not 23	207
**Embase**	
exp neoplasm/	4036884
exp hematopoietic stem cell transplantation/ or stem cell transplantation/ or stem cell mobilization/	99776
(cancer* or neoplas* or tumo?r* or malignan* or metasta* or oncogen* or oncolog* or hepatoma* or hepatoblastoma* or hepato-blastoma* or carcinoma* or adenoma? or adenocarcinoma? or adeno-carcinoma? or blastoma? or carcinosarcoma? or carcino-sarcoma? or leukemia? or leukaemia? or lymphoma? or melanoma? or mesenchymoma? or mesothelioma? or sarcoma? or thymoma?).tw,kw	4421296
(HSCT or ((h?ematopoietic or h?emato-poietic) adj3 (transplant* or sct or bct or mobili#ation)) or pbsct or pbct or psct or ((peripheral or pbsc or pbscs) adj3 transplant*) or autohct or autopbsct or autopbsc or autopsc or cbsct or ((autologous or auto-logous or auto or allogeneic or allo-geneic or homologous or homo-logous) adj hct)).tw,kw	60106
or/1-4	5189214
exp fear/	228473
exp Anxiety Disorder/	215071
(anxiet* or stress disorder* or psychological trauma* or ptsd or ptsds or moral injur* or post trauma* or posttrauma* or (stress* adj2 trauma*)).tw,kw	318527
(anxious* or fear or fears or fearful* or nervous* or angst or apprehensi* or panic* or phobic or phobia*).tw,kw	495919
or/6-9	892945
aromatherapy/ or moxibustion/ or reiki/ or shamanism/ or spiritual healing/ or exp acupuncture/ or exp biofeedback/ or qigong/ or breathing exercise/ or tai chi/ or yoga/ or movement therapy/ or hypnosis/ or guided imagery/ or meditation/ or mindfulness/ or psychodrama/ or kinesiotherapy/ or exp manipulative medicine/ or spiritual healing/ or art therapy/ or dance therapy/ or music therapy/	146944
((mind? adj1 body) or acupunctur* or acupotom* or electroacupunctur* or electro-acupunctur* or moxibustion or moxabustion or shaman* or witch* or sorcer* or radiesthesi* or aroma therap* or aromatherap* or (essential adj2 oil*) or bio-feedback* or myo-feedback* or psycho-physiologic feedback* or neuro-feedback* or biofeedback* or myofeedback* or psychophysiologic feedback* or neurofeedback* or alpha feedback* or brainwave feedback* or qigong or qi gong or ch'i kung or ((breath* or coping or respiratory) adj2 (exercise* or technique* or train*)) or relax* or hypnos#s or hypnotherap* or hypno-therap* or hypnotism or autogenic training or self-hypno* or imagery or reverie or laughter or laughing or meditat* or mindful* or psychodrama* or psycho-drama* or tai chi or tai ji or taiji or therapeutic touch* or healing touch* or reiki or yoga or energy therap* or ((musculoskeletal* or musculo-skeletal* or orthop?edic* or osteopathic* or spinal* or cervical* or lumbar*) adj2 manipulat*) or ((manipulat* or manual) adj2 therap*) or chiropractic* or soft tissue therap* or acupressure* or chih ya or shiatsu or shiatzu or zhi ya or bodywork* or massag* or rolfing or zone therap* or reflex therap* or reflexolog* or reflexotherap* or reflexo-therap* or manual lymph* or ((spiritual or faith or mental* or religion* or religious*) adj3 (healing or heal or healed or heals)) or prayer* or magic* or body align* or pilates or movement technique* or alexander technique* or feldenkrais or structural integration* or trager approach* or psycho-physical integration* or psychophysical integration* or mentastic* or ((art or music) adj3 therap*) or dance or dancing).tw,kw	357625
or/11-12	424914
randomized controlled trial/	534337
controlled clinical trial/	460240
exp randomization/	81310

Double Blind Procedure/	157804
Single Blind Procedure/	33834
((singl* or doubl* or trebl* or tripl*) adj (blind* or dumm* or mask*)).tw,kw	220973
PLACEBO/	329973
(placebo* or sham).tw,kw	389415
(randomized or randomised or randomly or rct).ab	1087493
trial.ti	262171
or/12-21	1578325
5 and 10 and 13 and 24	3754
(exp animal/ or nonhuman/) NOT exp human/	6009924
25 not 26	3678
**Scopus**	
TITLE-ABS-KEY(cancer* or neoplas* or tumor* or tumour* or malignan* or metasta* or oncogen* or oncolog* or hepatoma* or hepatoblastoma* or hepato-blastoma* or carcinoma* or adenoma* or adenocarcinoma* or adeno-carcinoma* or blastoma* or carcinosarcoma* or carcino-sarcoma* or leukemia* or leukaemia* or lymphoma* or melanoma* or mesenchymoma* or mesothelioma* or sarcoma* or thymoma* or HSCT or ((hematopoietic or hemato-poietic or haematopoietic or haemato-poietic) W/3 (transplant* or sct or bct or mobilization or mobilisation)) or pbsct or pbct or psct or ((peripheral or pbsc or pbscs) W/3 transplant*) or autohct or autopbsct or autopbsc or autopsc or cbsct or ((autologous or auto-logous or auto or allogeneic or allo-geneic or homologous or homo-logous) W/1 hct))	5343120
TITLE-ABS-KEY(anxiet* or (stress W/1 disorder*) or (psychological W/1 trauma*) or ptsd or ptsds or (moral W/1 injur*) or (post W/1 trauma*) or posttrauma* or (stress* W/2 trauma*) or anxious* or fear or fears or fearful* or nervous* or angst or apprehensi* or panic* or phobic or phobia*)	1663296
TITLE-ABS-KEY((mind W/1 body) or acupunctur* or acupotom* or electroacupunctur* or electro-acupunctur* or moxibustion or moxabustion or shaman* or witch* or sorcer* or radiesthesi* or aroma therap* or aromatherap* or (essential W/2 oil*) or bio-feedback* or myo-feedback* or "psycho-physiologic feedback" or neuro-feedback* or biofeedback* or myofeedback* or "psychophysiologic feedback" or neurofeedback* or "alpha feedback" or "brainwave feedback" or qigong or "qi gong" or "ch'i kung" or ((breath* or coping or respiratory) W/2 (exercise* or technique* or train*)) or relax* or hypnos#s or hypnotherap* or hypno-therap* or hypnotism or "autogenic training" or self-hypno* or imagery or reverie or laughter or laughing or meditat* or mindful* or psychodrama* or psycho-drama* or "tai chi" or "tai ji" or taiji or (therapeutic W/1 touch*) or (healing W/1 touch*) or reiki or yoga or (energy W/1 therap*) or ((musculoskeletal* or musculo-skeletal* or orthopedic* or orthopaedic or osteopathic* or spinal* or cervical* or lumbar*) W/2 manipulat*) or ((manipulat* or manual) W/2 therap*) or chiropractic* or "soft tissue therapy" or acupressure* or "chih ya" or shiatsu or shiatzu or "zhi ya" or bodywork* or massag* or rolfing or (zone N/1 therap*) or (reflex N/1 therap*) or reflexolog* or reflexotherap* or reflexo-therap* or (manual N/1 lymph*) or ((spiritual or faith or mental* or religion* or religious*) W/3 (healing or heal or healed or heals)) or prayer* or magic* or (body N/1 align*) or pilates or (movement N/1 technique*) or "alexander technique" or feldenkrais or "structural integration" or "trager approach" or "psycho-physical integration" or "psychophysical integration" or mentastic* or ((art or music) W/3 therap*) or dance or dancing)	39730
TITLE-ABS-KEY((clinic* w/1 trial*) OR (randomi* w/1 control*) OR (randomi* w/2 trial*) OR (random* w/1 assign*) OR (random* w/1 allocat*) OR (control* w/1 clinic*) OR (control* w/1 trial) OR placebo* OR (Quantitat* w/1 Stud*) OR (control* w/1 stud*) OR (randomi* w/1 stud*) OR (singl* w/1 blind*) or (singl* w/1 mask*) OR (doubl* w/1 blind*) OR (doubl* w/1 mask*) OR (tripl* w/1 blind*) OR (tripl* w/1 mask*) OR (trebl* w/1 blind*) OR (trebl* w/1 mask*)) AND NOT (SRCTYPE(b) OR SRCTYPE(k) OR SRCTYPE(p) OR SRCTYPE(r) OR SRCTYPE(d) OR DOCTYPE(ab) OR DOCTYPE(bk)	6440854

© 2020 Oberoi S et al. *JAMA Netw Open.*

OR DOCTYPE(ch) OR DOCTYPE(bz) OR DOCTYPE(cr) OR DOCTYPE(ed) OR DOCTYPE(er) OR DOCTYPE(le) OR DOCTYPE(no) OR DOCTYPE(pr) OR DOCTYPE(rp) OR DOCTYPE(re) OR DOCTYPE(sh))	
#1 AND #2 AND #3 AND #4	254

[a]The numbers of citations reflect the result of the initial search which was conducted in Feb, 2019. The search was updated in May 10, 2019

© 2020 Oberoi S et al. *JAMA Netw Open.*

eTable 2. Self-report Anxiety Assessment Scales Used in the Included Trials[a] (n=28)

Anxiety Scale Name	Number of Studies	Range of Scores	Higher Score Interpretation
Hospital Anxiety and Depression Scale-Anxiety	5	0-21	More anxiety
State Trait Anxiety Inventory	5	20-80	More anxiety
Profile of Mood States (anxiety subscale)	3	0-36	More anxiety
Symptom Checklist-90-Revised (anxiety scale)	3	NA	More anxiety
Self-Rating Anxiety Scale	2	20-80	More anxiety
Beck Anxiety Inventory	1	0-63	More anxiety
Depression, anxiety and stress scale	1	0-20	More anxiety
Generalized Anxiety Disorder Scale	1	0-21	More anxiety
Hamilton Anxiety Rating Scale	1	0-56	More anxiety
Personality Assessment Inventory	1	NA	More anxiety
Prostate Specific Anxiety Scale	1	NA	More anxiety
Visual Analog Scale	1	0-10	More anxiety

[a] Does not add to 28 as anxiety outcome is not reported by all the included studies
Abbreviations: NA-not available

© 2020 Oberoi S et al. *JAMA Netw Open.*

eTable 3. Self-report Depression Assessment Scales Used in the Included Trials[a] (n=28)

Depression Scale Name	Number of Studies	Range of Scores	Higher Score Interpretation
Hospital Anxiety and Depression Scale	5	0-21	More depression
Center for Epidemiological Studies Depression Scale	4	0-60	More depression
Profile of Mood States (depression subscale)	3	0-36	More depression
Symptom Checklist-90-Revised (depression scale)	3	NA	More depression
Self-Rating Depression Scale	2	20-80	More depression
Depression, anxiety and stress scale (DASSI 21)	1	0-42	More depression
Hamilton Rating Scale for Depression	1	0-50	More depression
Personality Assessment Inventory	1	NA	More depression
Personal Health Questionnaire Depression Scale	1	0-24	More depression

[a] Does not add to 28 as anxiety outcome is not reported by all the included studies
Abbreviations: NA-not available

© 2020 Oberoi S et al. *JAMA Netw Open.*

eTable 4. Self-report Health-related Quality of Life Assessment Scales Used in the Included Trials[a] (n=28)

Health-related Quality of Life Scale Name	Number of Studies	Range of Scores	Higher Score Interpretation
European Organization for Research and Treatment of Cancer QLQ-C30 (overall score)	4	0-100	Better quality of life
Functional Assessment of Cancer Therapy-Breast (FACT-B) (overall score)	2	NA	Better quality of life
36-Item Short Form Survey (SF-36) (general health)	2	0-100	Better quality of life
5-item World Health Organization Well-Being Index (WHO-5) (overall score)	2	0-100	Better quality of life
Functional Assessment of Cancer Therapy-Breast (FACT-G) (overall score)	1	NA	Better quality of life
Functional Assessment of Cancer Therapy-Breast (FACT-G) (overall score)	1	NA	Better quality of life

[a] Does not add to 28 as anxiety outcome is not reported by all the included studies
Abbreviations: NA-not available

© 2020 Oberoi S et al. *JAMA Netw Open.*

eFigure 1. Summary of the risk of bias of studies included in the systematic review (n= 28)

	Random sequence generation (selection bias)	Allocation concealment (selection bias)	Blinding of participants and personnel (performance bias)	Blinding of outcome assessment (detection bias)	Incomplete outcome data (attrition bias)	Selective reporting (reporting bias)	Other bias
Blaes 2016	?	?	−	−	?	?	?
Bower 2015	?	?	−	−	?	+	+
Branstrom 2010	+	?	−	−	−	+	+
Chambers 2017	+	+	−	−	?	+	+
Compen(a) 2018	+	+	−	−	?	+	+
Compen(b) 2018	+	+	−	−	?	+	+
Foley 2010	+	+	−	−	?	−	?
Franco 2019	+	?	−	−	?	−	?
Henderson 2013	?	?	−	−	?	−	?
Hoffman 2012	+	+	−	−	+	+	+
Jang 2016	?	?	−	−	+	+	+
Johannsen 2016	+	+	−	−	−	+	+
Johns 2015	+	?	−	−	+	?	+
Kenne 2017	+	+	−	−	+	+	+
Kingston 2015	+	?	−	−	?	+	+
Lengacher 2009	?	?	−	−	+	?	+
Lengacher 2016	+	?	−	−	+	+	?
Lerman 2012	?	?	−	−	−	?	+
Liu 2019	+	+	−	−	?	+	+
Lorca 2018	?	?	−	?	?	?	−
Monti 2006	+	?	−	−	?	+	+
Russell 2018	+	+	−	−	−	+	+
Schellekens 2016	+	+	−	−	−	+	+
Speca 2000	+	?	−	−	−	+	+
Vaziri 2017	?	?	−	−	−	+	?
Wurtzen 2013	+	+	−	−	?	?	+
Zernicke 2014	+	+	−	−	?	+	+
Zhang (a) 2017	+	?	−	−	+	+	+
Zhang (b) 2017	+	?	−	−	+	+	+

© 2020 Oberoi S et al. *JAMA Netw Open.*

eFigure 2. Funnel plot ''trim and fill'' technique assessing publication bias for short-term reduction in severity of anxiety. The x-axis represents the standard difference in means for the effect of mindfulness-based intervention. Estimated number of missing studies on left side = 8 (solid dots)

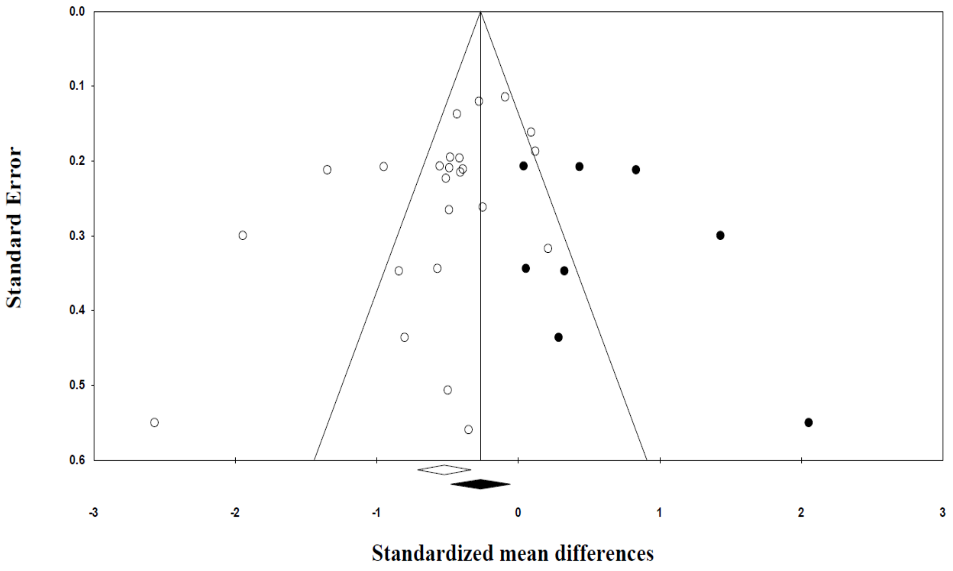

© 2020 Oberoi S et al. *JAMA Netw Open.*
